# Physiological and pharmacological roles of melatonin in the pathophysiological components of cellular injury after ischemic stroke

**DOI:** 10.3906/sag-2008-32

**Published:** 2020-11-03

**Authors:** Ertuğrul KILIÇ, Berrak ÇAĞLAYAN, Mustafa Çağlar BEKER

**Affiliations:** 1 Department of Physiology, School of Medicine, İstanbul Medipol University, İstanbul Turkey; 2 Regenerative and Restorative Medicine Research Center (REMER), Research Institute for Health Sciences andTechnologies (SABITA), İstanbul Medipol University, İstanbul Turkey; 3 Department of Medical Biology, International School of Medicine, İstanbul Medipol University, İstanbul Turkey

**Keywords:** Ischemic stroke, melatonin, free radicals, apoptosis, signaling, circadian rhythm proteins

## Abstract

Apart from its metabolic or physiological functions, melatonin has a potent cytoprotective activity in the physiological and pathological conditions. It is synthetized by the pineal gland and released into the blood circulation but particularly cerebrospinal fluid in a circadian manner. It can also easily diffuse through cellular membranes due its small size and lipophilic structure. Its cytoprotective activity has been linked to its potent free radical scavenger activity with the desirable characteristics of a clinically- reliable antioxidant. Melatonin detoxifies oxygen and nitrogen-based free radicals and oxidizing agents, including the highly toxic hydroxyl-and peroxynitrite radicals, initiating cellular damage. However, the cytoprotective activity of melatonin is complex and cannot be solely limited to its free radical scavenger activity. It regulates cellular signaling pathways through receptor– dependent and independent mechanisms. Most of these downstream molecules, such as PI3K/AKT pathway components, also contribute to the cytoprotective effects of melatonin. In this term, melatonin is a promising molecule for the treatment of neurodegenerative disorders, such as ischemic stroke, which melatonin reduces ischemic brain injury in animal models of ischemic stroke. It regulates also circadian rhythm proteins after ischemic stroke, playing roles in cellular survival. In this context, present article summarizes the possible role of melatonin in the pathophysiological events after ischemic stroke.

## 1. Introduction

Stroke is one of the leading causes of death and long-term disability worldwide [1] with increasing incidence rates in low and middle-income countries [2]. Disability due to a stroke incidence has significant emotional, social and economic burden on the patients and their society. It was suggested that deaths due to stroke cases will reach to 12 million and number of patients surviving from a stroke incidence will reach to about 70 million by the year 2030 [3].

Stroke cases are categorized into two groups: hemorrhagic and thrombotic (or ischemic) stroke. In hemorrhagic stroke, intracerebral or subarachnoid hemorrhage occurs in the brain due to the rupture of a cerebral blood vessel, but this type of stroke is less common (about 15% of all stroke cases in USA and Europe) worldwide [4,5]. On the other hand, ischemic stroke contributes to the majority of stroke cases and occurs when a cerebral artery is occluded by thrombus or thromboembolism, meaning that a blood clot may occur and cause a block in a small cerebral artery or a blot clot formed in a large vessel lodges in a small vessel in the brain. Due to the occlusion of the cerebral artery, blood supply to a certain brain region is restricted. Therefore, this event is called ischemia or ischemic stroke. 

Despite being a global problem with major social and economic burden on the society, treatment strategies are limited, and prevention measures are not enough [1]. Currently, patients survived from an ischemic stroke are treated with tissue plasminogen activator (tPA) that helps to relieve the obstruction in the blood vessels [6]. However, tPA has a very short therapeutic window which makes it impossible to use in the majority of the patients because of unfavorable actions of thrombolytics. Therefore, researchers have been in the search for a treatment option that can be used in patients who were diagnosed after the end of the therapeutic window of tPA or add-on treatment with thrombolytics. In this sense, it was shown that melatonin also reverses unfavorable actions of tPA [7] and, we will focus on the novel findings for the use of melatonin in ischemic brain injury in the present article.

The pathophysiologic mechanisms underlying ischemic stroke or ischemia/reperfusion injury in humans include loss of the prooxidant/antioxidant balance, excitotoxicity and related increase in intracellular Ca++ levels, dysfunction in mitochondrial processes, increased neuroinflammation and eventually apoptotic neuronal cell death [8].

## 2. Justification of use of melatonin in stroke treatment

The studies reporting the antioxidant effects of melatonin justified its use for treatment of ischemic injury in animal models [9]. Thereafter, further research provided evidence that melatonin is able to alleviate several of the detrimental effects of stroke-related pathophysiologic processes [10,11]. In addition, melatonin was shown to protect the integrity of blood-brain barrier [u291c], and functional recovery [17] with minimal, if any, side effects even in high doses [18]. Moreover, melatonin exerts neurological protection by reducing the cerebral inflammatory response, cerebral edema and brain-blood barrier permeability after ischemic stroke [19].

Although melatonin can be synthesized in a number of tissues, it is believed that the main production center may be the pineal gland due to the fact that melatonin’s protective effects are severely diminished by pinealectomy as reported in experimental model of stroke [20]. Melatonin synthesis in the pineal gland occurs in a circadian manner, in which the melatonin levels peak in the night-time, whereas in the day-time melatonin production is inhibited [21]. Secreted melatonin is believed to modulate the circadian clock mechanisms through the ubiquitin-proteasome signaling pathway. Several gene transcripts [22] and proteins [23] have been identified to be expressed in a circadian fashion. Of these genes, the six core clock genes are known as Per1 and Per2, Cry1 and Cry2, Clock and Bmal1 [24,25]. We have reported that melatonin regulates Bmal1 expression under normal conditions in vitro after hypoxia and in vivo after ischemic stroke [26]. Interestingly, in parallel with the highest blood concentrations of melatonin, the ischemic stroke incidence is lowest at the midnight hours in human [27]. In this term, we have indicated that tolerance to ischemic injury changes according to the time of day in which the injury occurs and the underlying mechanism of this tolerance includes circadian clock genes, specifically Bmal1, which is also regulated by the phosphorylation of AKT signaling pathway [24]. Consistently, Bmal1 expression is enhanced following melatonin treatment following ischemic brain injury in mice and this increase is blocked when the survival kinase AKT inhibitors are present [26]. In addition to the transcriptional control of Bmal1 gene, melatonin may also regulate clock genes by stabilizing the protein through the inhibition of the ubiquitin-proteasome system [28]. It has been speculated that melatonin can act as a proteasome inhibitor and we showed that melatonin inhibits the proteasome machinery by downregulating Nedd4-1 E3 ligase expression [29]. Collectively, these data suggest that melatonin may still have unelucidated roles in the body that promotes endogenous recovery systems.

## 3. Melatonin’s antioxidant effect

The experimental models in rodents focusing on the antioxidant effect of melatonin in stroke pathophysiology mainly involved in the ischemia/reperfusion. In this model, generally transiently middle cerebral artery occlusion (MCAo) is performed and melatonin is usually administered either at the onset of ischemia or reperfusion [8]. In the ischemia/reperfusion injury, a blood vessel is obstructed and blood supply to a certain brain region is restricted, causing immediate apoptotic and necrotic cell death in the ischemic core. The surrounding tissue, called the penumbra, has relatively higher levels of blood supply compared to the ischemic core, however, apoptotic cell death through the complex interplay of several mechanisms can be observed in the penumbra even several days after ischemic injury [8]. In order to resupply the ischemic brain tissue with oxygen, hyperbaric (HBO) and normobaric oxygen (NBO) treatments gained considerable interest due to the possibility of oxygen to diffuse through the blood-brain barrier to reach the injured brain tissue. However, conflicting literature exists for HBO treatment in transient or permanent MCAO models possibly because of increased free radical production upon effect of oxygen [30]. Even detrimental effects of HBO treatment were reported [31], but it should be kept in mind that the underlying reason for conflicting results may be partly because of the different timing of HBO treatment. Conversely, NBO therapy is an inexpensive and easy-to-access strategy that can be administered by simple facemasks. The favorable effects of NBO on the infarct volume and cell death were demonstrated in different models of brain injury [32–35]. However, the NBO treatment in these studies is usually started during the ischemia period or immediately at the onset of reperfusion. Although NBO can be beneficial in these models, this therapy can be only translated to patients who were admitted to hospitals with stroke symptoms that last for less than 12 h. In the light of these results, our group investigated the use of NBO treatment during reperfusion [12]. We also evaluated the combination of melatonin with NBO treatment. Our results indicated that melatonin potentiated the protective effect of NBO therapy in terms of infarct volume, brain swelling, neurological deficit score and DNA fragmentation [12].

Even though the most important step in rescuing the cells’ from apoptotic death in the penumbra is to remove the obstruction and resupply the blood flow (i.e. reperfusion), this results in oxidative damage due to the excess production of free radicals. The cells of the central nervous system (CNS) are already exposed to high amounts of free radicals in the physiological state. The adult human brain uses about 20% of the total oxygen intake even though it weighs only about 2% of the total body weight [36]. Not only brain produces the highest number of free radicals compared to any other organ in the body, but also it has high levels of polyunsaturated fatty acids which make it more prone to oxidative stress. Under normal conditions, the production of free radicals and antioxidant enzymes are kept in a delicate balance. If an imbalance occurs in favor of oxidants as a result of brain injury, the ability of the CNS cells to neutralize oxidants using endogenous antioxidant systems is overwhelmed and eventually inflammation and apoptotic cell death are observed. 

It is well-known that the free radicals including reactive oxygen species (ROS) and reactive nitrogen species (RNS), including superoxide anions (O2•−), hydrogen peroxide (H2O2), hydroxyl radicals (HO•) nitric oxide (•NO) and peroxynitrite (ONOO−) are mainly produced during the reperfusion phase of an ischemic stroke. The sources of these free radicals are diverse. For instance, nitric oxide is a signaling molecule that is involved in vasodilatation, neurotransmission and blood pressure maintenance and is synthesized by NO synthase (NOS) enzymes mainly in a calcium-dependent manner [37]. Three types of NOS enzymes were characterized as neuronal NOS (nNOS), inducible NOS (iNOS) and endothelial NOS (eNOS) in the central nervous system [38]. During ischemic brain injury, excess nitric oxide is generated, which in turn results in lipid peroxidation, energy depletion, and formation of highly neurotoxic oxidizing agent ONOO− [39]. Although the nitric oxide synthesized by the eNOS at the early phases of ischemic stroke is believed to provide protective effects, NO synthesis by iNOS and nNOS exacerbates the injury by activating the inflammatory mechanisms [40].

It has become clear that excessive free radical generation is a critical pathophysiological step in ischemic injury and regulates several other steps. This prompted researchers to investigate the possible use of chemical or biological molecules to reduce the generation or accumulation of free radicals. We and others have tested several antioxidant molecules, such as glutathione [41], vitamin E [42], vitamin C [43] or melatonin [20]. To the best of our knowledge, melatonin is the only antioxidant whose metabolites also have antioxidant capacities. In fact, the direct and indirect capacity of melatonin and its metabolites (c3OHM, AFMK and AMK) to scavenge free radicals is called the “antioxidant cascade” [44]. As the members of the antioxidant cascade of melatonin are all free radical scavengers, the detoxification capacity of melatonin was predicted to be up to 10 times more than any other antioxidant molecule [45]. Therefore, antioxidant capacity of melatonin has been studied in many ischemia/reperfusion injury models, including brain, kidney or heart. In fact, melatonin can interact with and detoxify the free radicals by donating an electron or a hydrogen atom in the injury models. In addition to its free radical scavenger activity, melatonin was also shown to upregulate antioxidant enzymes, such as superoxide dismutase (SOD), catalase or glutathione reductase (GR) [46]. Our group evaluated the effect of melatonin on the production of nitric oxide and our results demonstrate that melatonin treatment significantly downregulates nNOS and iNOS after ischemic brain injury [7,47].

## 4. Melatonin in mitochondria 

Considering the higher melatonin concentrations compared to blood levels were measured in rat liver mitochondria [48], it is not surprising to assume that high melatonin levels are required to protect the mitochondrial DNA from the continuous production of reactive oxygen species by the oxidative phosphorylation [49]. Because of its lipophilic structure, melatonin can easily pass through the biological membranes and accumulate in organelles, such as mitochondria and nuclei. Since the discovery of melatonin in bacteria and chloroplasts,it has been speculated that melatonin synthesis can also occur in the mitochondria [50]. Furthermore, enzymes (although not all) required for the synthesis of melatonin were detected in oocyte mitochondria [51]. These results strongly suggest that melatonin synthesized in the mitochondria is used as direct free radical scavenger and indirect antioxidant enzyme regulator in this organelle. Moreover, in a recent study, mitochondrial melatonin was shown to be secreted into the cytosol where it can bind to the receptors on the surface of the mitochondria [52]. It is proposed that this feature gives another advantage to melatonin in its role in mitochondrial homeostasis. This involves the restoration of the activity and expression of complexes I and IV which are decreased during ischemic injury. This in turn, reduces the electron leakage and prevents further damage to the organelle.

In addition, increased free Ca++ levels following ischemic brain injury are also responsible for mitochondrial dysfunction and formation of further free radicals. If these toxic effects are not neutralized, ATP production would be severely affected. Intriguingly, Xu et al. implicated that melatonin can control free Ca++ movement in the cytoplasm and protects the homeostasis of mitochondria [53]. 

## 5. Melatonin’s antiapoptotic effect

Loss of blood supply during ischemic brain injury depletes the cellular energy and results in the release of glutamate neurotransmitter into the extracellular space [8]. As a result, the transmembrane glutamate receptors (such as NMDA, AMPA or kainic acid receptors) and several channel and transporter proteins (such as TRPM2, TRPM7, NCX, ASICs, CaV1.2) are overactivated. Activation of these receptors is followed by an excessive Ca++ influx, either due to the release from mitochondria and endoplasmic reticulum or due to activation of plasma membrane proteins and lead to apoptotic cell death [54]. Of these receptors, overactivation of postsynaptic NMDA receptors by glutamate results in increased Ca++ load in the cytosol and mitochondria [55]. In this term, we used a combination treatment with an NMDA receptor inhibitor, memantine, and melatonin in a MCAo model and our results indicate that administration of melatonin/memantine combination significantly reduces the infarct volume, while improving the vascular leakage [56]. Furthermore, electrophysiological studies revealed that melatonin depresses NMDA receptor activity in the brain, possibly due to reduced nNOS activity [56,57].

In an attempt to rescue from Ca++ overload, the cells in the injured brain tissue try to reduce the high cytosolic Ca++ levels by storing Ca++ ions into mitochondria or endoplasmic reticulum. However, when Ca++ levels are increased in the mitochondrial matrix, free radical production is enhanced. Increased free radical accumulation disrupts mitochondrial membrane, results in permeabilization and depolarization followed by the release ofproapoptotic molecules, such as cytochrome c and apoptosis inducing factor (AIF) into the cytosol [u2934]. Andrabi et al. suggested that melatonin inhibits the cytochrome c release and decreases DNA damage in transient MCAO in rats [60]. Our results proved that melatonin improves neuronal survival by caspase-3 inhibition [16]. In addition, we showed that melatonin inhibits antiapoptotic Bcl-xL, while promoting the expression of proapoptotic Bax [12]. Downregulation of survival kinases in the injured brain tissue also contributes to apoptotic cell death. It is well-known that ischemia/reperfusion inhibits PI3K/AKT pathway. Our planar immunoassay analyses revealed that melatonin treatment results in the increased AKT phosphorylation especially at the Thr308 site of the activation loop via PDK1 and PTEN as well as decreased GSK-3α/β, and p53 phosphorylations [19], suggesting that neuroprotective activity of melatonin directly involves the activation of survival signaling pathways. Additionally, we have observed that melatonin treatment phosphorylates AMPKα, which is particularly activated by the reduced intracellular energy. AMPKα drives the cell to a catabolic state which this molecule mobilizes alternative energy sources, such as fatty acid oxidation, in order to supply ATP in the condition of ischemic stroke, suggesting also that melatonin may activate alternative collateral survival pathways [19].

## 6. Melatonin’s antiinflammatory effect

It has been shown that one of the early mechanisms of ischemic injury is the release of inflammatory cytokines, such as IL1β, IL6 and TNFα [61]. Following an ischemic attack in the brain, the free radicals are generated in high amounts. This excessive free radical production especially during the reperfusion phase also contributes to the disruption of the integrity of the blood-brain barrier and stimulates the infiltration of lymphocytes, neutrophils, monocytes, T cells, and macrophages to the injury site [62]. Simultaneously, resident microglial cells are activated, change their morphology (deramification) and start to release proinflammatory cytokines. Microglial activation following ischemia also results in increased proliferation and accumulation in the penumbra region. Microglial activation seems to have a dual role in the pathophysiology of stroke. During the course of ischemia/reperfusion, microglial cells are believed to switch from an antiinflammatory state to proinflammatory phenotype [63]. Melatonin treatment was shown to inhibit the proinflammatory shift of microglial cells through the regulation of SIRT1 and STAT3 [64,65]. Moreover, TLR4 activates NF-κB after stroke, resulting in the secretion of inflammatory molecules (IL1β, IL6 and TNFα). Melatonin decreases the secretion of inflammatory mediators by downregulating NF-κB, while promoting Nrf2 upregulation [66]. 

## 7. Roles of melatonin receptors in stroke treatment

Studies indicated increased melatonin levels can be observed as early as in the first 10 minutes of intraperitoneal or subcutaneous application, indicating that melatonin can easily pass through the blood-brain barrier [67]. Melatonin is also able to diffuse through cellular and organelle membranes due its small size and lipophilic structure [68]. On the other hand, melatonin has two G protein-coupled transmembrane receptors; MT1 and MT2 (earlier names Mel1a and Mel1b) which are ubiquitously found in almost all cells in the body [69]. It has been proposed that melatonin exerts its neuroprotective effects on the brain by both receptor-independent or receptor dependent mechanisms [70]. Models of acute and chronic ischemia have been used to investigate the role of MT1/MT2 receptors in ischemic brain injury by using melatonin receptor agonists, such as ramelteon [71,72], agomelatine [u2937] or Neu-P11 [u2938] and whether it is involved in melatonin’s antioxidant, antiapoptotic or antiinflammatory activity should be further investigated. Interestingly, MT3 knockout mice were less susceptible to menadione toxicity [80], suggesting that the inhibition of this receptor may have protective effects. Moreover, knockdown of MT3 by RNA interference in vitro resulted in enhanced expression of antioxidant enzymes [81], while overexpression of MT3 resulted in excessive production of reactive oxygen species [82]. It was shown that melatonin is able to inhibit this receptor at nanomolar levels in which antioxidant effects were documented [83]. Therefore, it is tempting to speculate that MT3 inhibition by melatonin is involved in melatonin’s protective effects on the pathophysiological outcomes of brain injury not only by increasing the expression of antioxidant enzymes, but also by providing resistance to oxidative stress.

Moreover, the nuclear receptors RZR/ROR from the retinoic acid receptor superfamily were proposed as nuclear binding sites for melatonin [84]. RZR/ROR expression was demonstrated in the brain, and in the pineal gland [69]. ROR receptors were shown to induce the expression of several clock genes, including Clock, Cry or Bmal1 [85–87] by binding to retinoic acid-related orphan receptor response elements (ROREs) in the promoter region. In parallel with these data, cyclic expression of ROR mRNAs were noted in different tissues, suggesting a circadian function possibly under the control of melatonin. However, whether these receptors play a part in the protective mechanisms induced by melatonin should be further investigated.

## 8. New roles for melatonin: regulation of circular RNA in ischemic injury

Recently, noncoding RNAs including microRNAs, long noncoding RNAs, and circular RNAs have gained considerable attention as regulatory molecules. Recent studies indicated that circular RNAs (circRNAs) are found abundantly in the brain and are involved in the embryonic development [88]. CircRNAs are enriched in specific brain regions such as cerebellum, cortex, striatum, olfactory bulbs, and hippocampus. Since they are circular, these types of noncoding RNAs are more resistant to digestion. CircRNAs can be made from introns, coding or noncoding exons, or from both exons and introns by a process called “back-splicing” [89]. Zhang et al. suggested that because the circRNAs are formed by the circularization of skipped exons, formation of circRNA can cause the downregulation of its parental gene by using up the pre-mRNA molecules [90]. In addition to their regulatory roles in the brain, circRNAs are also associated with neurological disorders, including, but not limited to, ischemia/reperfusion injury, traumatic brain injury, and Alzheimer’s disorder [91]. In a study performed in acute ischemic stroke patients to profile the changes in circRNA, 3 circRNAs have been proposed as diagnostic and predictive biomarkers for stroke [92]. Significantly altered circRNAs in a transient MCAO model in C57BL/6J mice were characterized, and bioinformatics data suggested that all these circRNAs possess binding sites for microRNAs [93]. However, the exact pathophysiological mechanisms that they have a role in are not fully elucidated. It has been predicted that melatonin may be involved in the regulation of circRNAs as the circRNA prolife in the pineal gland has been altered in a mouse model of Alzheimer’s disease [94]. In fact, a recent study reported that melatonin exerts protective effects through the regulation of CircRIC3/miR-204-5p/DPP4 signaling in calcific aortic valve disease [95]. Therefore, we hypothesized that melatonin also regulates circRNAs in ischemia/reperfusion injury, however, these circRNAs should be further investigated in future studies. 

## 9. Prophylactic use in high risk individuals

Moreover, we reported that lower endogenous melatonin concentrations were associated with increased injury after transient ischemic stroke in pinealectomized rats and when those deprived animals were given exogenous melatonin, injury size could be reduced [20]. It has been shown that melatonin levels are reduced in elderly compared to young adults; therefore, aged population is more prone to serious ischemic injury [96]. In addition, accumulation of other risk factors, such as cardiovascular disorders, diabetes, obesity or hypertension with ageing increases the risk of ischemic injury incidence [97,98]. In animal models, we demonstrated that prophylactic uses, and delayed uses of melatonin successfully protects the brain from the ischemic injury [u2960]. In conclusion, prophylactic doses could be considered in the elder population in order to compensate for the reduced melatonin levels due to the calcification of pineal gland and to promote the endogenous repair mechanisms against stroke or other neurodegenerative diseases.
